# Neuro-Oncology and Positron Emission Tomography: “Just Can’t Get Enough”

**DOI:** 10.3390/cancers15194739

**Published:** 2023-09-27

**Authors:** Barbara Muoio, Vittoria Espeli, Giorgio Treglia

**Affiliations:** 1 Division of Medical Oncology, Oncology Institute of Southern Switzerland, Ente Ospedaliero Cantonale, 6501 Bellinzona, Switzerland; barbara.muoio@eoc.ch (B.M.); vittoria.espeli@eoc.ch (V.E.); 2 Division of Nuclear Medicine, Imaging Institute of Southern Switzerland, Ente Ospedaliero Cantonale, 6501 Bellinzona, Switzerland; 3 Faculty of Biomedical Sciences, Università della Svizzera Italiana, 6900 Lugano, Switzerland; 4 Faculty of Biology and Medicine, University of Lausanne, 1011 Lausanne, Switzerland

Imaging has a pivotal role in neuro-oncology for the management of primary and secondary brain tumors. To this regard, positron emission tomography (PET) is increasingly used to supplement magnetic resonance imaging (MRI) in the management of patients with brain tumors, including gliomas, meningiomas, central nervous system lymphomas and brain metastases for specific clinical settings [1,2,3].

PET imaging is performed using different radiopharmaceuticals which evaluate several biological processes including different metabolic pathways or receptor expression [1,2,3]. Furthermore, current PET tomographs include a morphological component, computed tomography (CT) or MRI, therefore hybrid imaging (PET/CT or PET/MRI) is performed through these tomographs providing both functional and morphological information. The morphological component of hybrid imaging methods is useful for the anatomical localization of areas of abnormal radiopharmaceutical uptake at PET imaging and for attenuation correction of PET images [1,2,3].

An umbrella review published in 2019 which summarizes evidence-based data on PET using different radiopharmaceuticals in brain tumors showed a good diagnostic performance of PET using different radiopharmaceuticals for specific indications in brain tumors and a significant prognostic value [4]. Recent literature data reinforce these findings about PET in neuro-oncology.

Gliomas are the most common neuro-epithelial tumors of the central nervous system in adults; most of gliomas are highly malignant tumors with high rate of recurrence and a dismal prognosis. Advanced imaging methods including PET with different radiopharmaceuticals are useful to differentiate among tumor progression and treatment-related changes in gliomas; this differential diagnosis has prognostic and therapeutical implications [5]. Interestingly, a recent meta-analysis comparing perfusion-weighted MRI and PET with a variety of radiopharmaceuticals showed no diagnostic superior imaging technique in differentiating between tumor progression and treatment-related changes in gliomas. As no imaging technique was found to be diagnostically superior, the local level of expertise is hypothesized to be the most important factor for diagnostically accurate results [6]. A recent systematic review which considered the 2021 WHO classification of brain tumors highlighted the usefulness of PET imaging with different radiopharmaceuticals in distinguishing among tumor recurrence and radiation necrosis in adult-type diffuse gliomas revealing high heterogeneity among studies [7]. Beyond these indications, PET imaging using amino acid radiopharmaceuticals could be used in diagnosis and grading of gliomas, for a guide to biopsy and for surgical and radiotherapy planning; in particular, an integration of PET imaging during resection and radiotherapy planning providing more meaningful information than standard MRI could increase survival in patients with gliomas [8].

Meningioma is the most common intracranial tumor, typically a slow-growing neoplasm that forms from the meninges and it can be incidentally detected by PET imaging using different radiopharmaceuticals [9]. Furthermore, due to overexpression of somatostatin receptors in most of meningiomas, PET using radiolabeled somatostatin analogues may be used for detection of meningioma tissue, delineation of meningioma extent for resection planning or radiotherapy planning, differentiation of tumor progression/disease relapse from post-treatment changes and for theranostic applications [10,11]. Incorporation of somatostatin receptor PET into routine management of meningioma has the potential to overcome some limitations of standard neuroimaging [10,11].

Primary central nervous system lymphoma (PCNSL) is an uncommon neoplasm with a poor prognosis. PET with fluorine-18 fluorodeoxyglucose has a defined role for the management of PCNSL in immunocompetent and immunocompromised patients. This imaging method is useful for assessing the disease spread at the time of diagnosis. In addition, it can provide valuable information for differential diagnosis and outcome prediction, and for treatment response assessment [12].

Brain metastases can be also evaluated by PET with different radiopharmaceuticals, and in particular amino acid tracers. Along with MRI, the gold standard for diagnosis of brain metastases, PET is a useful complementary imaging method technique for evaluation of selected indications in patients with brain metastases. Current evidence highlights the relevant role of amino acid PET radiotracers in brain metastases, in particular for the differentiation among tumor progression/disease relapse after treatment and treatment-related changes [13].

PET/CT is currently the most used hybrid imaging modality used for the evaluation of brain tumors. However, there is growing evidence that a multimodal approach using hybrid PET/MRI can achieve several improvements in the diagnostics of brain tumors [14].

Beyond the technology advancements of PET tomographs, future perspectives about PET imaging in neuro-oncology are represented by novel PET radiopharmaceuticals evaluating different biological processes of brain tumors [15,16,17,18,19] and the use of artificial intelligence and machine learning methods applied to PET imaging to ameliorate the diagnostic process, the prognostic evaluation and treatment response assessment [20].

Even if increasing evidence-based data about PET imaging in brain tumors are available, high-quality studies are still warranted (“we just can’t get enough”) to strengthen the role of this imaging method in neuro-oncology.
